# Grain Production Space Reconstruction and Its Influencing Factors in the Loess Plateau

**DOI:** 10.3390/ijerph19105876

**Published:** 2022-05-12

**Authors:** Zhangxuan Qin, Xiaolin Liu, Xiaoyan Lu, Mengfei Li, Fei Li

**Affiliations:** 1 College of Urban and Environmental Science, Northwest University, Xi’an 710127, China; qinzhangxuan@stumail.nwu.edu.cn (Z.Q.); liuxl0110@163.com (X.L.); 202032513@stumail.nwu.edu.cn (X.L.); 15684197130@163.com (M.L.); 2 Yellow River Institute of Shaanxi Province, Xi’an 710127, China

**Keywords:** grain production space, reconstruction pathway, spatiotemporal evolution, influencing factors, Loess Plateau

## Abstract

Grain production space, ecological service space and urban–rural development space are the classifications of land systems from the perspective of the dominant function of the land system. Grain production space reconstruction concentrates on the principal contradictions of land system changes, and is the key to exploring the transformation of land system. Therefore, the pathways, process and influencing factors of grain production space reconstruction in the Loess Plateau of Chian from 1980 to 2018 was explored from three dimensions of quantity–quality–spatial pattern in this study. Results showed that the quantity of grain production space showed a slight downward trend with a net decrease of 9156 km^2^ between 1980 and 2018, but its total quality showed a fluctuating growth trend under rain-fed conditions. Due to the intensification of human activities, grain production space was gradually fragmented, and the distribution tended to be decentralized, and the shape gradually became regular. Meanwhile, both the quantity and quality gravity center of grain production space moved to the northwest by 8.32 km and 86.03 km, respectively. The reconstruction of grain production space in the Loess Plateau was mainly realized through four pathways: Grain for Green, Urban Expansion, Deforestation and Reclamation, and Land Consolidation. The grain production space was mainly reconstructed through the pathway of Grain for Green after 2000. The four reconstruction pathways were the result of a combination of natural environment and socio-economic factors, but influencing factors had different strengths and directions for each reconstruction pathway. From the perspective of social economy–land use–ecological environment coupling, in order to maintain the sustainable development of the land systems, it is necessary to reduce the trade-offs of the functions of land systems as much as possible and strive to coordinate the relationship among grain production, ecological protection and high-quality development.

## 1. Introduction

Land systems, coupled social economy–land use–ecological environment, possessed the multiple functions [1,2], such as ensuring grain security, providing ecological services, promoting economic prosperity, etc. The development and evolution of land systems had a profound impact on the realization of sustainable goals [3,4,5]. According to the dominant function of land systems, land systems can be divided into grain production space, urban–rural development space and ecological service space [6,7]. However, it was difficult for land systems to achieve all sustainable development goals simultaneously, and there were trade-offs among different functions [8,9,10], and resulted in the global land systems undergoing changes characterized by grain production space reconstruction, urban–rural development space expansion and ecological service space contraction [11,12,13]. The grain production space reconstruction, referred to by the changes in the quantity, quality and spatial pattern of grain production space, and caused by the subjective trade-offs of different stakeholders on land system functions, was closely related to the expansion of urban–rural development space and the contraction of ecological service space, concentrating on the main contradictions of the changes in the land system. Research on grain production space reconstruction can not only deepen the understanding of land system changes, but also help coordinate the contradictions among land system functions and promote the realization of sustainable development goals.

Since the 1980s, the quantity of global grain production space has shown an insignificant increase [14,15], but there were significant regional differences [16]. The loss of grain production space mainly occurred in economically developed and densely populated areas, resulting from Urban Expansion [17,18,19], or in ecologically fragile areas through policy guidance to protect the ecological environment, and gradually converted cultivated land into forest or grassland [20,21]. In areas with relatively slow economic development, different stakeholders reclaimed land to pursue economic benefits and ensure grain security, and grain production space increased slightly [22,23]. The quality of grain production space was a complex internal attribute [24], and natural features (fertility, structure, texture and stability etc.) and human activities (fertilization, irrigation, and crop rotation etc.) directly, indirectly, synergistically or antagonistically affected the quality of grain production space [25,26,27]. Hence, it was difficult to establish a unified standard to evaluate the quality of grain production space [28]. Climate changes such as CO_2_ increasing, temperature rising [29] and an increase in extreme weather [30], as well as human activities such as Land Consolidation [31], Urban Expansion [32] and intensive utilization of grain production space [33], profoundly affected the quality reconstruction. Under the severe influence of human activities, fragmentation was intensified, shape was complicated and spatial distribution tended to be unstable for grain production space [34,35,36]. In the context of global warming and the squeeze of grain supply and demand, the gravity center of grain production space had a tendency to expand toward high latitudes [14,37]. Scholars paid more attention to the changing rules of landscape pattern and spatial heterogeneity of quantitative changes of grain production space, but there was little study of the spatial pattern evolution of its quality. Natural environment factors were the basis of grain production space reconstruction, and controlled the direction of grain production space reconstruction [38,39]; socio-economic factors were the core driving force for grain production space reconstruction, and determined speed and intensity of the reconstruction [40,41,42]; often the driving role of socio-economic factors was more significant [43,44]. These studies analyzed characteristics of grain production space reconstruction from different dimensions, and provided a series of models and analysis frameworks for analyzing grain production space reconstruction. However, it was necessary to establish a comprehensive framework to systematically analyze the reconstruction of grain production space in terms of quantity, quality and spatial pattern.

The Loess Plateau was one of the most serious soil erosion areas and the most fragile ecological environment in China and the world. Since the reform and opening up, climate change and human activities such as cultivated land reclamation, Urban Expansion, and returning farmland to forests and grasses, significantly reconstructed the grain production space in the Loess Plateau. Therefore, this study selected the Loess Plateau as a case study, and analyzed the characteristics and influencing factors of grain production space reconstruction by developing a systematic analysis framework, aimed at solving the following issues:

(a) What was the principal pathway for the reconstruction of grain production space in quantity, quality and spatial pattern?

(b) What were the factors that affected the reconstruction pathways in the Loess Plateau?

The research results will provide a new research perspective on rational land systems management, coordinate grain production, ecological protection and high-quality economic development, and provide a theoretical basis of ensuring the sustainable and intensive development of grain production space and grain security in the Loess Plateau.

## 2. Material and Methods

### 2.1. Analysis Framework

Economic development, grain production and ecological services were the basic functions provided by land systems for human society. According to the dominant function of land systems, it is divided into grain production space, ecological service space and urban–rural development space. Cultivated land that mainly provides grain production function is defined as grain production space. Construction land that promotes economic development is divided into urban–rural development space. Although forest and grassland provide a series of agricultural products such as fruits and pastures for human beings, their more important function is to maintain ecological services. Therefore, forest and grassland are divided into ecological service spaces according to their dominant function.

**Figure 1:**
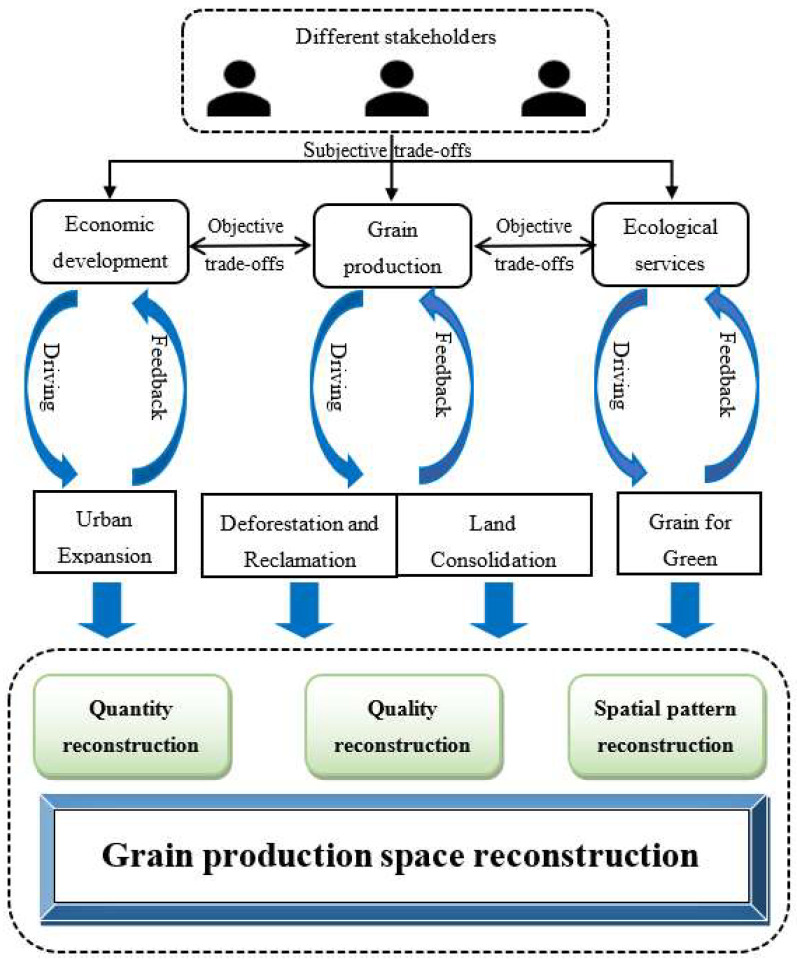
The systematic analysis framework of grain production space reconstruction.

Different degrees of trade-offs exist between the dominant function of land systems. The subjective trade-offs of different stakeholders in land system functions drive the changes of land use. Meanwhile, different reconstruction pathways of grain production space led to objective trade-offs between land system functions. Hence, grain production space was reconstructed in the transmission process of subjective trade-offs and objective trade-offs. Economic development promoted Urban Expansion, which occupied a large number of cultivated land and affected grain production. In order to ensure grain security, Deforestation and Reclamation as well as Land Consolidation restored a large amount of grain production space. However, Deforestation and Reclamation led to the degradation of the natural environment, Grain for Green converted cultivated land into forests or grasslands to improve ecological services function. In addition, grain production space reconstruction was reflected in three aspects: quantity, quality and spatial pattern, and there were significant differences in the impact of different pathways on grain production space reconstruction. Therefore, this study assumed there was only one reconstruction pathway in grain production space, then compared its reconstruction characteristics with the initial year, next obtained the principal pathway for the reconstruction of grain production space in quantity, quality, and spatial pattern (Figure 1). Based on the systematic analysis framework of grain production space reconstruction, this study was organized by the following:

(i) Assessing the characteristics of the quantity, quality and spatial pattern reconstruction of grain production space;

(ii) Determining the principal pathway for the reconstruction of grain production space in quantity, quality and spatial pattern;

(iii) Defining the influencing factors of each reconstruction pathway.

### 2.2. Study Area

**Figure 2:**
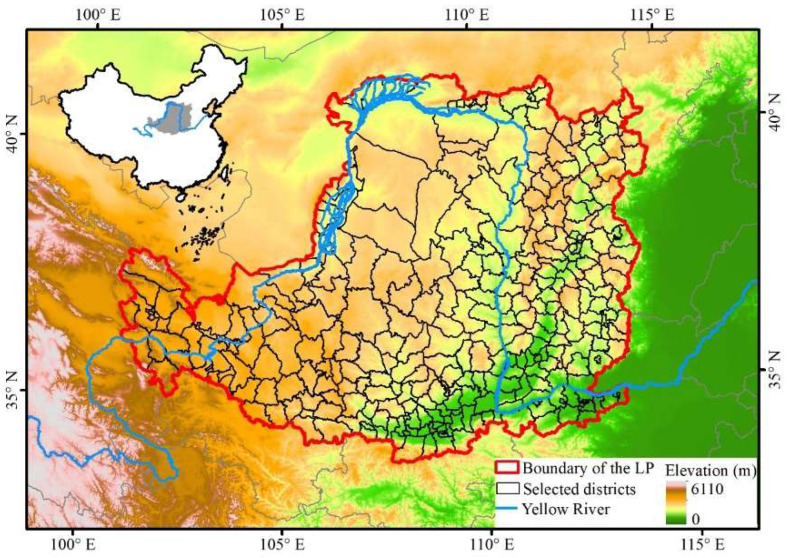
Overview of the study area.

The Loess Plateau (33°41′ N~41°16′ N, 100°54′ E~114°33′ E) of China covers an area about 6.35 × 10^5^ km^2^, which is the largest loess geomorphic unit in the world. The Loess Plateau covers 7 provinces, including Shanxi, Shaanxi, Henan, Inner Mongolia, Gansu, Ningxia, and Qinghai (Figure 2). It is situated in a semi-arid and semi-humid climate zone, with concentrated precipitation in summer, broken terrain, loose soil, and frequent soil erosion. Hence, it is one of the regions with the most serious soil erosion and vulnerable ecological environment in the world. As a national key construction area of the “Grain for Green Project”, the implementation of ecological projects such as Grain for Green Project had reconstructed the grain production space of the Loess Plateau in terms of quantity and pattern. The Loess Plateau is a typical rain-fed agricultural area, and grain production is highly dependent on regional climatic conditions. In the past half century, the climate of the Loess Plateau showed a trend of warming and drying. Temperature rise was a specific feature in the context of global warming, and the decrease in precipitation was a local feature. The change of climate not only profoundly restored and rebuilt vegetation, but also reconstructed the quality of grain production space. Therefore, in this study, we selected 332 districts and counties in the Loess Plateau with significant reconstruction of grain production space as the study area.

### 2.3. Data Sources

The data used in this research mainly included land use data, meteorological data, soil data, topographic data, river data, transportation network data and socioeconomic data (Table 1). 

### 2.4. GAEZ Model

This research used the Global Agro-Ecological Zone (GAEZ) model of grain production potential under rain-fed conditions (only considering the impact of climate on crop yield) to reflect the quality of grain production space. Wheat and corn were mainly considered in the calculation process, because these two crops yield accounted for about 80% of the total grain yield in the Loess Plateau. The GAEZ model was developed jointly by the Food and Agriculture Organization of the United Nations (FAO) and Institute of International Applied Systems Analysis (IIASA). The model estimated the climatological suitability of a crop and then calculated crop potential yield by using a progressively limiting method. For the detailed computation process of GAEZ, please refer to Global Agro-ecological Zones. The GAEZ model is widely used in the world, because of the availability basic data, the simplicity of the calculation process and more accurate calculation results [45,46,47].

### 2.5. Landscape Pattern Index

Landscape pattern index was used to analyze landscape pattern reconstruction of grain production space, and selected Patch Density (PD), Patch Area_Mean (AREA_MN), Aggregation Index (AI), Landscape Division Index (DIVISION), Fractal Dimension Index_Area-Weighted Mean (FRAC_AM) to reflected the characteristics of the area, distribution, and shape reconstruction in grain production space.

### 2.6. Gravity Center Model

The concept of the gravity center came from physics. The gravity center was considered a balance point, because it was relatively balanced in the front, back, left and right directions of the force [48]. Gravity center model can objectively reflect the spatial difference and change trajectory of a certain element in the regional development process [49,50]. Population center gravity model, economic gravity center model [51] and production gravity center model [49] were commonly used in geography. Based on this, in order to describe the overall change trend and spatial variation characteristics of grain production space, this study attempted to construct the gravity center model of the quantity and quality of grain production space according to the gravity center model theory. The basic model was as follows:

(1) $$X_{ti}=\frac{\sum_{i=1}^{n}\left(C_{t}\times X_{i}\right)}{\sum_{i=1}^{n}C_{t}}$$

(2) $$Y_{ti}=\frac{\sum_{i=1}^{n}\left(C_{t}\times Y_{i}\right)}{\sum_{i=1}^{n}C_{t}}$$

where *X_ti_* and *Y_ti_* are the horizontal and vertical coordinates of the grain production space in year *t* in area *i*; *C_t_* is the area in year *t* in area *i*; *X_i_* and *Y_i_* are the longitude and latitude of the geometric center of gravity in area *i*.

### 2.7. Spatial Econometric Regression Model

The book “Spatial Econometrics: Methods and Models” published by Anselin (1988) had become a milestone in the development of spatial econometrics. Anselin pointed out that “Almost all spatial data have the characteristics of spatial dependence or spatial autocorrelation” [52]. Anselin incorporated spatial factors neglected in previous econometric models into the model, established econometric model considering spatial factors [53]. As grain production space reconstruction usually showed the spatial autocorrelation characteristics, this study used the spatial econometric regression model to measure the influencing factors of grain production space reconstruction. The spatial econometric model used in this study was mainly a spatial constant coefficient regression model that incorporates spatial effects (spatial correlation and spatial differences), including Spatial Lag Model (SLM) and Spatial Error Model (SEM).

The SLM mainly discusses whether each variable has a diffusion phenomenon (spillover effect) in a region. The spatial lag model (SLM) can be specified as:

(3) Y=ρWy+Xβ+ε

where *Y* is the dependent variable; *X* is the independent variable; *W* is the spatial weight matrix and *Wy* is a vector of spatial lag dependent variable. *ρ* is a spatial regression coefficient that reflects the spatial dependence of the sample observations, *β* is the parameter vector of *X* and *ε* is the model error term.

The SEM describes spatial disturbance correlation and spatial overall correlation. It can be written as:

(4) Y=Xβ+ε

(5) ε=λWε+μ

where *Y, X, β* and W are the same as those in Equation (3), *λ* is the autoregressive parameter, *μ* is the random error vector of the normal distribution and *ε* is the regression residual.

## 3. Results

### 3.1. Quantity Reconstruction of Grain Production Space

From 1980 to 2018, the proportion of grain production space in the Loess Plateau in land system remained above 30%, and the overall trend was slightly declining with a net decrease of 9156 km^2^ (Table 2).

**Figure 3:**
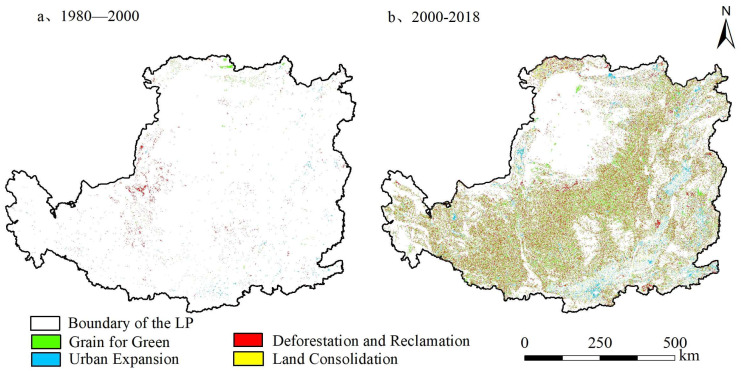
The quantity reconstruction of grain production space in the Loess Plateau.

Although the overall quantity of grain production space showed a slight declining trend, its internal reconstruction was frequent (Figure 3). The quantity reconstruction of grain production in the Loess Plateau had been increasing over time, mainly occurred after 2000. Since 2000, contribution rates of Grain for Green, Deforestation and Reclamation, Urban Expansion and Land Consolidation to grain production space reconstruction were 42.80%, 39.42%, 8.32% and 4.23%, respectively. Therefore, Grain for Green after 2000 was the principal pathway that affected the quantity reconstruction of grain production space.

### 3.2. Quality Reconstruction of Grain Production Space

**Figure 4:**
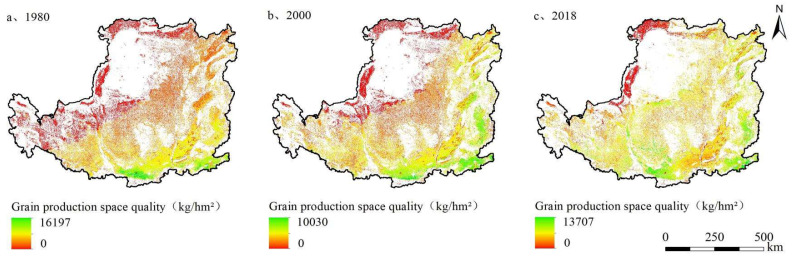
Spatial distribution of grain production space quality in the Loess Plateau from 1980 to 2018.

The average quality of grain production space was about 3896.11 kg/hm^2^, and its total quality showed a fluctuating growth trend under rain-fed conditions in the Loess Plateau in the past 40 years. Due to differences in temperature, precipitation, soil types, etc., the spatial distribution of grain production space quality showed a decreasing distribution characteristic from southeast to northwest (Figure 4).

Assuming that there was only one reconstruction pathway in grain production space, and comparing the influence of four reconstruction pathways on quality reconstruction, the results showed that (Table 3): Grain for Green and Land Consolidation were reconstruction pathways to increase the quality of grain production space, while Deforestation and Reclamation as well as Urban Expansion were pathways that affected the quality deterioration of grain production space. However, the pathway of Grain for Green after 2000 had the greatest impact on quality reconstruction of grain production space in the Loess Plateau.

### 3.3. Pattern Reconstruction of Grain Production Space

**Figure 5:**
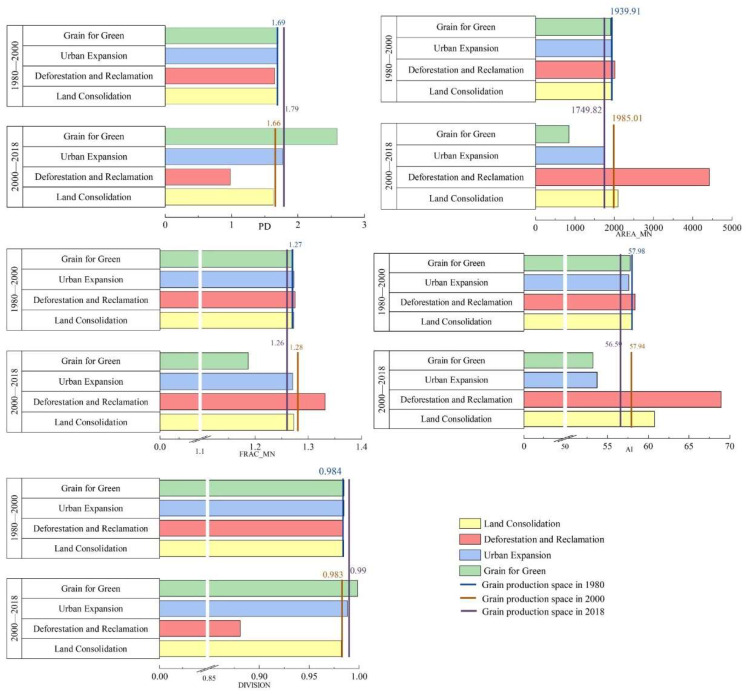
The landscape pattern reconstruction of grain production space in the Loess Plateau.

From 1980 to 2018, PD of grain production space in the Loess Plateau was on an increasing trend, but AREA_MN has decreased overall (Figure 5). It showed that the large patches were continuously divided into small patches, and the area of grain production space was more fragmented. Fragmentation degree increased more obviously after 2000. The changes in AI and DIVISION were more obvious after 2000, and showed an obvious reverse development trend (Figure 5). It indicated that the spatial distribution of grain production space tended to be scattered in the Loess Plateau after 2000. FRAC_AM showed an increasing trend from 1980 to 2000, but it represented a decreasing trend from 2000 to 2018. The change range was between 1.25 and 1.28, and the change was relatively small. FRAC_AM was far from reaching the upper limit of 1.5, indicating that the shape of the grain production space was complex, but there was a trend toward regularization (Figure 5). Therefore, since 1980, the impact of human activities on land use changes became more and more intense, which leading to grain production space area was more fragmentation, the distribution tended to be decentralized, and the shape became more regular.

Assumed there was only one reconstruction pathway in grain production space, and compared with the initial year landscape pattern index. We found that reconstruction pathways after 2000 had a more profound impact on landscape pattern reconstruction than 1980–2000, and Grain for Green after 2000 was the principal pathway for grain production space reconstruction (Figure 5). Grain for green led to an increase in PD and a decrease in AREA_MN, which intensifying the fragmentation of grain production space; made AI decrease and Division increase, which promoting the spatial distribution to be more scattered; and caused a decrease in the value of FRAC_AM, which motivating the shape more regular.

**Figure 6:**
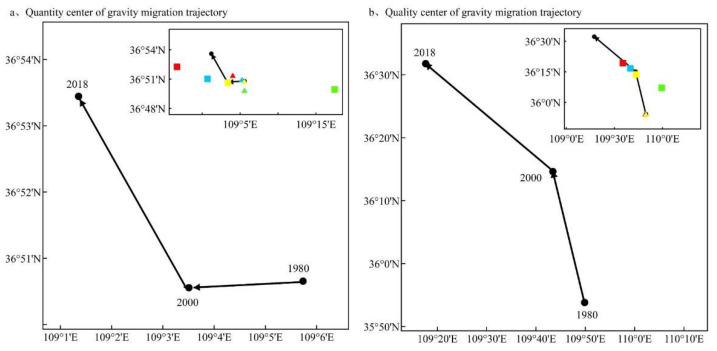
The quantity (**a**) and quality (**b**) gravity center of grain production space in the Loess Plateau from 1980 to 2018. Green—Grain for Green, blue—Urban Expansion, red—Deforestation and Reclamation, yellow—Land Consolidation; triangle represents 1980–2000, square represents 2000–2018. The combination of color and shape represents the influence of a single reconstruction pathway in two stages on the change of the grain production space quantity and quality center of gravity.

From 1980 to 2018, the gravity center of grain production space quantity in the Loess Plateau moved 8.32 km to the northwest. The gravity center of grain production space quality had the same movement direction, but it moved farther and reached 86.03 km (Figure 6). Compared with 1980–2000, reconstruction pathways after 2000 had a greater impact on the movement of the gravity center (Figure 6). The pathway of Grain for Green from 2000 to 2018 had the greatest impact on the gravity center of quantity and quality movement in grain production space. Compared with 2000, this pathway caused the quantity and quality gravity center to move southeast by 20.79 km and 29.71 km, respectively.

### 3.4. Driving Factors for Grain Production Space Reconstruction

Firstly, we performed a series of statistical tests. Four reconstruction pathways (Grain for Green, Urban Expansion, Deforestation and Reclamation as well as Land Consolidation), nine independent variables (average altitude, average slope, river density, average temperature change, average precipitation change, total population change, GDP change, highway mileage change and railway mileage change) Tol > 0.1 and VIF < 10, indicating that there is no collinearity between independent variables. All data followed a normal distribution. There was an inevitable endogeneity between reconstruction pathways and influencing factors, which can be ignored in the study of geographical influencing factors [54,55,56]. Then, we chose the suitable model for influencing factor analysis. We calculated the Moran’s I value and White test of grain production space reconstruction pathways in the Loess Plateau. The results showed that (Table 4) there was a significant positive spatial autocorrelation for different reconstruction pathways, and Urban Expansion and Land Consolidation had heteroscedasticity. Hence, a traditional linear regression model cannot be used to explore the influencing factors of regional grain production space reconstruction, and it is necessary to establish the spatial econometric model to analyze the influencing factors. Next, we performed LMlag, LMerror, R-LMlag and R-LMerror tests. As shown in Table 4, LMlag tests and R-LMlag tests were more significant for the reconstruction pathways for Grain for Green, Deforestation and Reclamation as well as Land Consolidation, but the statistics of LMerror tests and R-LMerror tests were more significant for Urban Expansion. In terms of the fitting effect detection of the regression model, the smaller the R^2^ and Log Likelihood (LogL) and the greater the Akaike Information Criterion (AIC) and Schwartz Criterion (SC), the better the model fitting effect (Table 5). Therefore, SLM was employed in the estimation of Grain for Green, Deforestation and Reclamation as well as Land Consolidation, and SEM was employed in the estimation of Urban Expansion.

The pathway of Grain for Green was significantly correlated with average slope, highway mileage change, average precipitation change, and average altitude (Table 5), indicating that the reconstruction mainly occurred in areas with steeper slopes, lower terrain, increased precipitation, and more new highways. Among the above driving factors, average slope had the most significant impact of this reconstruction. In order to curb soil erosion and solve a series of ecological problems, the Loess Plateau actively responded to the national policy of Grain for Green. In total, 64.35% grain production space with a slope above 25° was transformed into forests or grasslands. Grain for Green also mostly occurred in areas where precipitation increased, the increase of precipitation provided abundant water for vegetation restoration. Convenient transportation made it easier for farmers to go out, and an increasing number of farmers planted trees instead of grain on their own grain production space when they went out to find work.

The pathway of Urban Expansion had a high correlation with GDP change, highway mileage change, railway mileage change, average slope and average precipitation change (Table 5). It showed that Urban Expansion mostly occurred in areas with rapid economic development, rapid transportation network improvement, gentle slopes, and reduced precipitation. Urban expansion had the most affected driving factors among the four reconstruction pathways. In particular, GDP change only had a significant effect on Urban Expansion, but had no significant effect on the other three reconstruction pathways, it indicated that areas with superior economic conditions were more likely to be transformed into urban–rural development space. Meanwhile, low slope provided the nature basis for the reconstruction pathway.

The pathway of Deforestation and Reclamation was highly correlated with average slope and highway mileage change in a county (Table 5). In the early stage of reform and opening up, grain production had a higher priority in production and life. However, constrained by lower capital and technology input, expanding grain production scale was an inevitable choice to ensure grain security. Under this background, the government encouraged farmers to deforest and reclaim on steep slopes. With the increase of highway mileage, the convenience of grain export increased income, which stimulating farmers to continue to reclaim.

The pathway of Land Consolidation had a significant correlation with the average slope and the change of the transportation network (Table 5). This pathway mainly occurred after 2000, the developed transportation network was conducive to the migration of rural population to the cities. From 2000 to 2018, the rural population of the Loess Plateau decreased by about 11 million, and the proportion of the rural population in the total population dropped from 74.6% to 57.6%. The migration of rural population promoted the non-agriculturalization and part-time employment of rural labor, and the rural population decreased, so a large number of homesteads were left idle. Under the guidance of policies (dynamic balance between farmlands and construction lands, balance of cultivated land occupation and compensation etc.), a large number of idle land resources had been revitalized, and effectively increasing the space for grain production. In addition, lower slope was conducive to agricultural production activities, and a large amount of idle land was more likely to be reclaimed.

In general, the four pathways of grain production space reconstruction in the Loess Plateau were the result of the combined effects of natural environment and socio-economic driving factors. We adjusted the sample period from 2000 to 2018 and performed regression analysis using the same model as before, and obtained regression results similar to those in Table 5. The spatial econometric regression model results at the county level indicated that all factors had significant impacts on four pathways, except for river density, average temperature change and total population change. Average slope and highway mileage change had a significant effect on all reconstruction pathways, but their direction of action and intensity were different. Moreover, average altitude and GDP change only had a significant impact on one reconstruction pathway (Table 5). The spatial econometric regression analysis results of Deforestation and Reclamation was not ideal, because the reconstruction pathway was too concentrated in the Loess Plateau; analysis of the other three reconstruction pathways was ideal.

## 4. Discussion

Since the reform and opening up, under the dual pressures of reducing supply and increasing demand of grain production space, Deforestation and Reclamation was always the most common, effective and direct pathway to replenish grain production space [11,57]. Large-scale Deforestation and Reclamation had aggravated soil erosion, and a series of ecological and environmental problems had become prominent in the Loess Plateau. In order to slow down soil erosion and restore vegetation, the government implemented Grain for Green Project since 1999. Different from the slow response of other countries’ land use changes in national policies, the Loess Plateau responded to national policies actively and quickly [58] and made a great contribution to improve China’s forest coverage. From 2000 to 2018, the quantity of Grain for Green accounted for 42.8% of the quantity reconstruction of grain production space, which was the main pathway of quantity reconstruction. Although the pathway of Grain for Green, it has reduced the number of grain production space, and has caused poor quality grain production space that is withdrawing from the planting field, and agricultural production factors are concentrated in higher-quality areas, thereby increasing the quality of grain production space by 298.61 kg per hectare. Under the unified guidance of the national plan, Grain for Green promoted the more regular shape and the more concentrated distribution of grain production space. Poor quality grain production space reclaimed on steep slopes was returned to forest or grassland. The original grain production space was divided severely, which intensified the fragmentation of grain production space. As the number of Grain for Green after 2000 was large and concentrated in the southwest and north of the Loess Plateau, this pathway had a great impact on the quantity and quality gravity center of grain production space, and the gravity center of grain production moved 20.79 km and 29.71 km to the southeast, respectively. Therefore, Grain for Green after 2000 was the principal pathway for grain production space reconstruction in quantity, quality and spatial pattern in the Loess Plateau.

We studied the influencing factors of grain production space reconstruction in the Loess Plateau, and found that the reconstruction was the result of a combination of natural environment and socio-economic factors. In the influencing factors analysis, river density, average temperature change and total population change did not show a direct correlation. Water source was an important factor restricting grain production in the Loess Plateau, but with the advancement of science and technology, sprinkler irrigation, drip irrigation and other technologies had solved the problem of water shortage in grain production. Therefore, river density did not show a significant correlation in the process of grain production space reconstruction. In the past hundred years, climate warming was not a local feature [59], so temperature changes had not triggered the quantity reconstruction in the Loess Plateau. Grain production space reconstruction was due to the grain pressure caused by population increase rapidly, but the main reconstruction areas were spatially misplaced with densely populated areas. Therefore, in the analysis of the factors affecting the reconstruction of grain production space, river density, average temperature change and total population change did not show a direct correlation.

**Figure 7:**
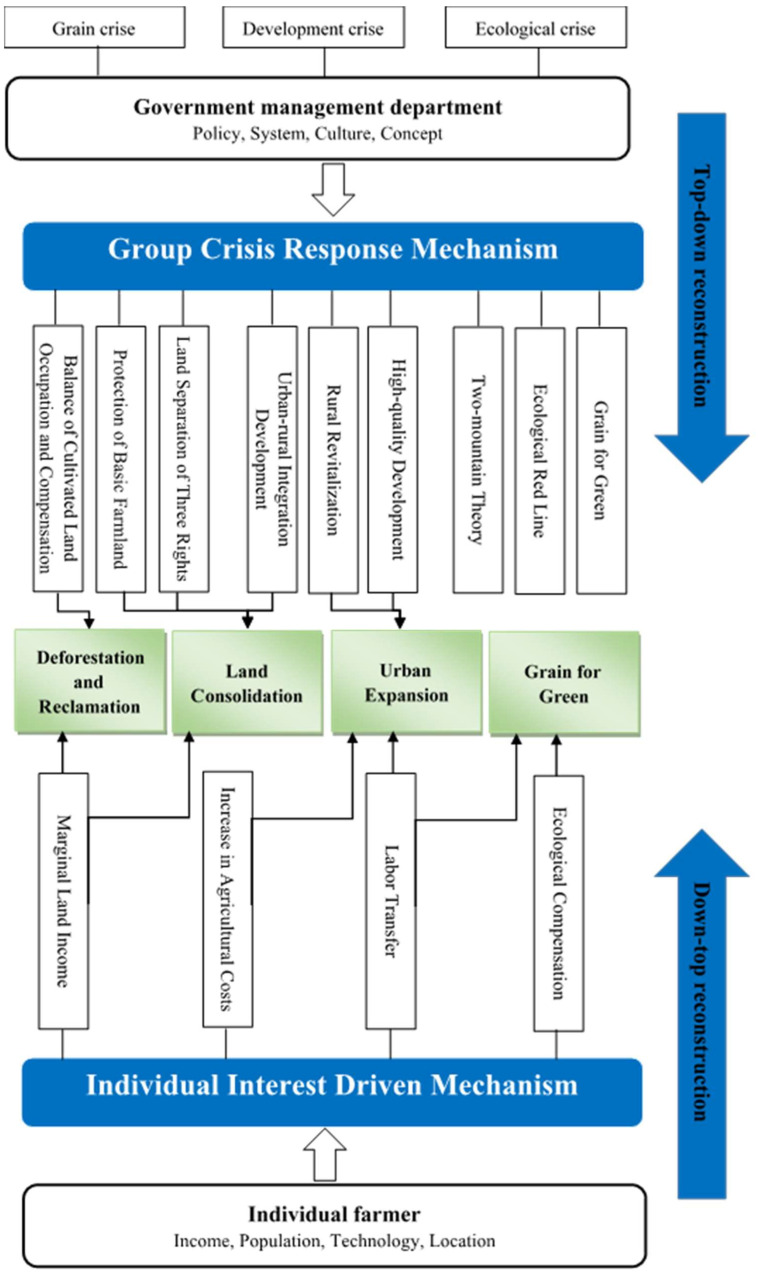
Grain production space reconstruction driving mechanism.

System, policy, economy, location and other factors drive the subjective trade-offs of land system functions by different stakeholders, and changing the land use [60]. The subjective trade-offs of government management departments and individual mainly drive grain production space reconstruction through two mechanisms: group crisis response and individual interest-driven (Figure 7). System changes and policy adjustments play a leading role in the process of grain production space reconstruction [61,62]. Ecological protection policies and land management systems can affect directly the grain production space reconstruction, and macro-control policies and regional development policies can affect indirectly the reconstruction [54,63,64]. The dissemination of cultural knowledge and the transformation of value concepts could drive the reconstruction of grain production space through the process of transmission and feedback between different levels of politics, economy, and society [65]. In the context of rising agricultural production costs and higher income from going out to work, after weighing their benefits, stakeholders actively change land use to obtain higher income, thereby reconstructing grain production space. Factors such as the deterioration of the rural ecological environment and the increase in employment opportunities in cities have led to rural population migration and structural adjustment, which inevitably affects the reconstruction of grain production space. Hence, population change is the most direct driving force for grain production space reconstruction [62,66]. The spatial heterogeneity of location can determine the spatial evolution of geographical elements. Being close to big cities is susceptible to the diffusion of capital, technology, talents, transportation and other factors, which drives the development of secondary and tertiary industries, and weakens the position of agriculture in economic activities. Superior location conditions can promote the transformation of grain production space into urban–rural development space [67]. The promotion of agricultural technology could advance the intensive use of high-quality land and abandon poor-quality land, so less land will produce more goods and services [66]. Therefore, system, policy, income, population, culture, concept, technology and location affect the reconstruction of grain production space by different stakeholders.

The group crisis response mechanism (Figure 7) is when grain, development or ecological crises occur on limited land resources, endangering the survival and development of human society, and the government or management department will take measures to deal with the crises by the group—which is the top-down grain production space reconstruction [68]. In the early stages of economic development or in ecologically fragile areas, expanding the scale of grain production is an advisable choice to ensure grain security. Therefore, the government or management department will encourage Deforestation and Reclamation. The natural environment determines the limited amount of land suitable for agricultural production. It is necessary to achieve agricultural intensification and reduce the demand for land. In addition, increasing population requires more production and living land. Therefore, the government and management departments expand urban–rural development space and increase economic benefits through urban expansion and land planning. When Urban Expansion threatens grain security, the government may formulate different policies (basic farmland preservation areas, dynamic balance between farmlands and construction lands, etc.) to slow down the shrinkage of grain production space. Meanwhile, while the government continues to increase technical input, a large number of idle lands are reclaimed under the guidance of policies. Hence, grain production space is restructured. Deforestation and Reclamation and Urban Expansion have destroyed the stability and integrity of the ecosystem, forcing government to take measures such as Grain for Green to restore and protect the ecosystem, and grain production space is reconstructed accordingly.

Individual interest-driven mechanism reconstruct the grain production space from down to top, which means that land users adjust their land use in order to pursue their own maximum economic benefits under the background of changes in land rent, land benefits and opportunity costs (Figure 7). Individuals always pursue the maximization of their own economic benefits, while ignoring ecological and social benefits. Therefore, different stakeholders operate and use land resources in a way that they believe can generate the greatest economic benefits [69]. When the income of the original land management mode is lower than the new management mode (or the opportunity cost of agricultural production) due to economic, policy, technology and other reasons, grain production space will be reconstructed. The increase in population brings about an increase in the demand for grain. Driven by economic benefits, farmers deforest and reclaim on steep slopes. Individual in areas with good location, flat terrain and suitable climate tend to increase capital and technology input, and transform grain production space into urban–rural development space to gain more benefits. Social and economic development bring more employment opportunities and income to individuals, and increases the opportunity cost of agricultural production. Driven by individual economic interests, farmers abandon their original grain production space for population migration and restore to natural vegetation in original grain production space [70,71]. In addition, the national ecological compensation policies also lead farmers to return farmland to forests or grasses spontaneously to obtain a higher income than farmland.

Many scholars had analyzed the reconstruction process of grain production space from different dimensions. Zhang et al. [6] analyzed the grain production space reconstruction in China since the reform and opening up from the three dimensions of “quantity-quality- spatial pattern”. Based on the research of Zhang et al., this study took the Loess Plateau as the research area, not only analyzed the grain production space reconstruction from the three dimensions and identified the principal pathway affecting the reconstruction, but also determined the influencing factors of each reconstruction pathway. However, this research also has some limitations. First of all, grain production space refers to the land with grain production as its main function. This research defines cultivated land as grain production space, but in reality, the land has multi-functional attributes, cultivated land and grain production space are not completely coupled. In future studies, we may strive to adopt more rigorous land division methods, and scientifically and objectively divide grain production space, ecological service space and urban–rural development space. Secondly, based on different stakeholders, the group crisis response mechanism and individual interest-driven mechanism that affect grain production space reconstruction is proposed. In our next study, we will select a series of representative evaluation indicators to quantitatively study the group crisis response mechanism and individual interest-driven mechanism, in order to analyze the driving mechanism of grain production space reconstruction more objectively, comprehensively and scientifically. In the future, a similar analysis framework can be used to explore the reconstruction characteristics of other spaces in different regions and scales, and identify its principal reconstruction pathway.

## 5. Conclusions

The land systems have multiple functions, and there is a trade-off/synergy between different functions. Grain production space reconstruction concentrates on the main contradictions of the land system changes. Therefore, this study analyzed the reconstruction process and influencing factors of grain production space in the Loess Plateau. The goal was to answer the question: “what was the principal pathway for the reconstruction and what were the factors that affected the reconstruction pathways of grain production space?” The results showed that the quantity of grain production space in the Loess Plateau declined, but the total quality improved between 1980 and 2018. The spatial pattern of grain production space tended to be fragmented, scattered and regular. The gravity center of grain production space’s quantity and quality moved to the northwest; the quality gravity center moved farther. Grain for Green after 2000 was the principal pathway in quantity, quality and spatial pattern reconstruction of grain production space. The reconstruction pathways of Grain for Green, Urban Expansion, Deforestation and Reclamation as well as Land Consolidation were the result of a combination of natural environment and socio-economic factors, but influencing factors had different strengths and directions for each reconstruction pathway in the Loess Plateau. Therefore, there is no absolute synergy between grain production, ecological services and economic development. It is necessary to reduce the trade-offs of land system functions as much as possible, strive to coordinate the relationship among grain production, ecological protection and high-quality economic development, and promote the sustainable and intensified development of grain production space in the Loess Plateau.

## Figures and Tables

**Table 1:** Brief introduction of the data set used in this study.

Data Type	Key Indicators	Data Source	Temporal Attribute
Land use data	Grain production space Urban-rural development space Ecological service space	Chinese Academy of Sciences Resource and Environmental Science Data Center	1980; 2000; 2018
Meteorological data	Precipitation Mean maximum temperature Mean minimum temperature Wind speed Relative humidity Rainfall day Solar radiation	China Meteorological Administration	Monthly; 1980–2018
Topographic data	DEM	U.S. Space Shuttle Radar Topography Mission	2008
River data	River density	National Basic Geographic Information System	2017
Transportation network	Highway mileage Railway mileage	Atlas of China and Atlas of China Transportation published by Sinomap press	1981; 2019
Socioeconomic data	Total population Gross domestic product	China Statistical Yearbook (The missing statistics were inferred from the GM (1,1) model)	1980; 2018

**Table 2:** Changes in quantity of grain production space in the Loess Plateau from 1980 to 2018.

Time	Quantity/km^2^	Proportion of Land System
1980	204,331	32.70%
2000	206,262	33.01%
2018	195,175	31.24%

**Table 3:** The influence of the reconstruction pathway of grain production space on quality reconstruction of grain production space in the Loess Plateau.

Stage	Reconstruction Pathway	Difference from the Average Quality in Initial Year (kg/hm^2^)
1980–2000	Grain for Green	16.61
	Urban Expansion	−14.16
	Deforestation and Reclamation	−39.59
	Land Consolidation	0.03
2000–2018	Grain for Green	298.61
	Urban Expansion	−62.30
	Deforestation and Reclamation	−155.07
	Land Consolidation	21.59

**Table 4:** Spatial dependence test of the reconstruction pathway of grain production space in the Loess Plateau.

Statistical Tests	Grain for Green	Urban Expansion	Deforestation and Reclamation	Land Consolidation
Moran’s I	0.6241 ***	0.3051 ***	0.6062 ***	0.4542 ***
White test	0.091 *	0.000 ***	0.223	0.008 ***
Lagrange Multiplier (lag)	182.375 ***	30.23 ***	175.957 ***	129.322 ***
Robust LM (lag)	16.462 ***	1.434	22.989 ***	27.328 ***
Lagrange Multiplier (error)	168.935 ***	43.76 ***	153.276 ***	105.439 ***
Robust LM (error)	3.022 *	14.963 ***	0.308	3.444 *

Note: * and *** indicate statistical significance at 10% and 1% levels.

**Table 5:** The spatial regression analysis results of grain production space reconstruction in the Loess Plateau.

Driving Factors	Grain for Green	Urban Expansion	Deforestation and Reclamation	Land Consolidation
Average altitude	−0.039	0.001	−0.020	0.002
Average slope	9.649 ***	−1.633 ***	6.742 ***	−0.868 ***
River density	6.684	−8.526	−91.759	0.629
Average temperature change	−12.481	1.766	−7.083	−2.901
Average precipitation change	0.262 **	−0.063 **	0.157	−0.013
Total population change	−0.232	0.055	0.285	0.016
GDP change	0.008	0.034 ***	−0.016	−0.009
Highway mileage change	0.486 ***	0.067 ***	0.445 ***	0.024 **
Railway mileage change	0.358	0.188 ***	0.302	0.060 **
W-Y	0.686 ***		0.701 ***	0.727 ***
Lambda		0.439 ***		
R^2^	0.650	0.395	0.628	0.496
LogL	−2131.41	−1550.65	−2110.84	−1424.08
AIC	4284.82	3121.32	4243.69	2870.16
SC	4326.68	3159.37	4285.54	2912.02

Note: ** and *** indicate statistical significance at 5% and 1%.

## Data Availability

The data that support the findings of this study are available from the corresponding author upon reasonable request.
